# Using the Medical Research Council framework and public involvement in the development of a communication partner training intervention for people with primary progressive aphasia (PPA): Better Conversations with PPA

**DOI:** 10.1186/s12877-021-02561-8

**Published:** 2021-11-15

**Authors:** Anna Volkmer, Aimee Spector, Kate Swinburn, Jason D. Warren, Suzanne Beeke

**Affiliations:** 1 Division of Psychology and Language Sciences, University College London, London, UK; 2 Dementia Research Centre, Department of Neurodegenerative Disease, UCL Institute of Neurology, University College London, London, UK

**Keywords:** Primary progressive aphasia, Speech and language therapy, Intervention, Conversation, Co-production, Consensus

## Abstract

**Background:**

Primary progressive aphasia is a language-led dementia resulting in a gradual dissolution of language. Primary progressive aphasia has a significant psychosocial impact on both the person and their families. Speech and language therapy is one of the only available management options, and communication partner training interventions offer a practical approach to identify strategies to support conversation. The aim of this study was to define and refine a manual and an online training resource for speech and language therapists to deliver communication partner training to people with primary progressive aphasia and their communication partners called Better Conversations with primary progressive aphasia.

**Methods:**

The Better Conversations with primary progressive aphasia manual and training program were developed using the Medical Research Council framework for developing complex interventions. The six-stage development process included 1. Exploratory review of existing literature including principles of applied Conversation Analysis, behaviour change theory and frameworks for chronic disease self-management, 2. Consultation and co-production over 12 meetings with the project steering group comprising representatives from key stakeholder groups, 3. Development of an initial draft, 4. Survey feedback followed by a consensus meeting using the Nominal Group Techniques with a group of speech and language therapists, 5. Two focus groups to gather opinions from people with PPA and their families were recorded, transcribed and Thematic Analysis used to examine the data, 6. Refinement.

**Results:**

Co-production of the Better Conversations with primary progressive aphasia resulted in seven online training modules, and a manual describing four communication partner training intervention sessions with accompanying handouts. Eight important components of communication partner training were identified in the aggregation process of the Nominal Group Technique undertaken with 36 speech and language therapists, including use of video feedback to focus on strengths as well as areas of conversation breakdown. Analysis of the focus groups held with six people with primary progressive aphasia and seven family members identified three themes 1) Timing of intervention, 2) Speech and language therapists’ understanding of types of dementia, and 3) Knowing what helps. These data informed refinements to the manual including additional practice activities and useful strategies for the future.

**Conclusions:**

Using the Medical Research Council framework to develop an intervention that is underpinned by a theoretical rationale of how communication partner training causes change allows for the key intervention components to be strengthened. Co-production of the manual and training materials ensures the intervention will meet the needs of people with primary progressive aphasia and their communication partners. Gathering further data from speech and language therapists and people living with primary progressive aphasia and their families to refine the manual and the training materials enhances the feasibility of delivering this in preparation for a phase II NHS-based randomised controlled pilot-feasibility study, currently underway.

**Supplementary Information:**

The online version contains supplementary material available at 10.1186/s12877-021-02561-8.

## Background

The number of people living with dementia worldwide continues to rise, estimated at around 50 million at present with nearly 10 million new cases each year [1]. Of these, perhaps a half a million people worldwide and several thousand in the United Kingdom have Primary Progressive Aphasia (PPA): a group of language-led dementias associated with Frontotemporal Dementia and Alzheimer’s disease [2]. PPA presents as an insidious dissolution of language skills with relative sparing of other cognitive functions [2]. At present there are three internationally recognised PPA variants; people with semantic variant experience a gradual loss of word meanings affecting both comprehension and naming, people with logopenic variant PPA present with difficulties in word retrieval and processing of complex sentences, and people with non-fluent agrammatic variant PPA demonstrate effortful, distorted articulation of speech sounds (apraxia) and/or an agrammatism [3]. Each variant presents with a distinct neuroanatomical distribution of atrophy and underlying neuropathology [2, 3]. Though it constitutes only a small proportion of the total dementia burden, PPA is of disproportionate clinical importance because it tends to strike people in older midlife with devastating impact on occupational and social functioning and because it presents a number of unique challenges not well met by conventional models of aphasia and dementia management.

People with PPA report increasing social isolation and reduced confidence as a result of their worsening communication difficulties [4]. More than one third of people with PPA experience depression and symptoms of anxiety are not uncommon. These likely impact directly on reports of reduced quality of life amongst people with PPA [5]. Spouses of people with PPA report a long trajectory of change, even prior to diagnosis. This results in feelings of loss of relationship and meaningful social interaction, increasing dependency of their spouse with PPA on them for communication, and overwhelming responsibility [6].

The research literature on speech and language treatment approaches for people with PPA is developing. The majority of research has focused on impairment-focused interventions that aim to maintain or improve the person’s ability to use words [7, 8]. Many people with PPA disengage from such naming therapies due to the frustration of practising individual words they will inevitably lose as the disease progresses [9]. More recently there has been a growing focus on functional communication interventions for PPA, which aim to support a person to execute an activity or participate in a life situation [10]. A systematic review of these diverse interventions identified two key shared components; building on existing strategies, and practising strategies with a communication partner [10].

Despite barriers to therapy access, such as a lack of awareness of the role of the speech and language therapist in PPA, and restrictive service criteria, the number of people with this condition being referred to speech and language therapy is increasing [11]. In contrast to a research focus on naming therapies, in clinical practice speech and language therapists prioritise communication partner training (CPT) interventions for people with PPA and their communication partners (CPs; who may be anyone close to the person such as spouses, family members or friends) [11, 12].

CPT interventions for stroke and dementia have arisen from studies of conversation between people with communication disorders and their CPs. This research demonstrates that both people with dementia and aphasia draw on areas of retained strength, such as gesture, to maintain interactional flow [13–15]. Some CPs are seen to facilitate conversational interaction, for example through giving time, but can equally expose their partners’ difficulties by using barrier behaviours, for example, test questions (to which they already know the answer, a pedagogic behaviour used with children). CPT interventions aim to change conversation behaviours, enhancing conversational skill and confidence, and reducing barriers to facilitate the flow of natural conversation [16]. CPT interventions result in improved quality of life and wellbeing for people with dementia, and improved competency in their CPs [17].

Many speech and language therapists report delivering CPT to people with PPA and describe using resources developed for stroke aphasia or brain injury related communication difficulties [12]. CPT has a growing evidence base in stroke aphasia [16, 18] and delivers positive changes in the conversation skills of people with aphasia as well as their CPs [19, 20]. However, CPT approaches in stroke aphasia are not designed to meet the needs of people with progressive communication difficulties. Currently there are only case study reports of CPT for people with PPA [21, 22]. There is some suggestion of increased communicative effectiveness as a result, however, it is difficult to attribute these gains to CPT due to the fact that individuals were concurrently participating in additional interventions. Thus, there is a clinical need to develop a CPT intervention designed to meet the needs of people with PPA and their families [6, 23, 24].

To our knowledge there has been no specific research undertaken asking people with PPA and their families what interventions are important or need to be developed. People with PPA have written about their general experiences of speech and language therapy and the value of developing “a wide range of personalized strategies that continually evolve as the disease progresses” [25]. Spouses report a need to develop practical approaches to deal with communication difficulties and maintain a close bond with their loved ones [6]. These issues are more likely to be met by tailored interventions, that build capacity by helping them to adjust and reframe their communication over time [6]. Speech and language therapists themselves have identified a need to engage family who are motivated to understand how they can best support their loved ones [26]. Therefore, gathering ideas and contributions of people living with PPA, often described as Public Involvement, is important to ensuring an intervention will meet their needs. Public Involvement is defined by the UK Standards for Public Involvement as research that is carried out with members of the public rather than to them [27]. These standards include ensuring that people are involved as early as possible, and that participation is made accessible. Co-production is defined as a way of working where people (service users) and providers work together to reach a collective outcome [28]. The aim of this study was to work with people with PPA and their families, from the beginning, to co-produce a CPT intervention to meet their needs.

Ensuring strict standardisation is unlikely to be appropriate given the need to tailor CPT to an individual’s needs but understanding what causes the change so this can be identified and strengthened in the development process is key. This complex intervention, with its multiple interacting components, such as working with both a person with PPA and their CP, will be difficult to evaluate. The Medical Research Council provide a framework for developing and evaluating complex interventions [29]. The guidance outlines the importance of preliminary development and testing of an intervention’s procedures prior to piloting and evaluation. This paper therefore describes how the Medical Research Council framework was used to develop Better Conversations with PPA (BCPPA), a 4-session, manualised, CPT intervention to help people with PPA and their CPs to identify and practice strategies to reduce barriers (such as interjecting when a person may not have finished) and increase facilitators in conversations (such as giving more time). A manual and an online training resource for speech and language therapists, hosted on a life-learning platform at UCL, were developed to enable speech and language therapists to deliver the intervention. In line with stages 1 and 2 of the Medical Research Council Framework the underlying theory and proposed mechanisms of change for the BCPPA program will be described as well as primary research which informed the co-production of the manual and online training resource.

### Aim

To use the Medical Research Council framework for developing complex interventions to define and refine a manual and an online training resource for speech and language therapists to deliver BCPPA to people with PPA and their CPs.

## Methods

**Fig. 1:**
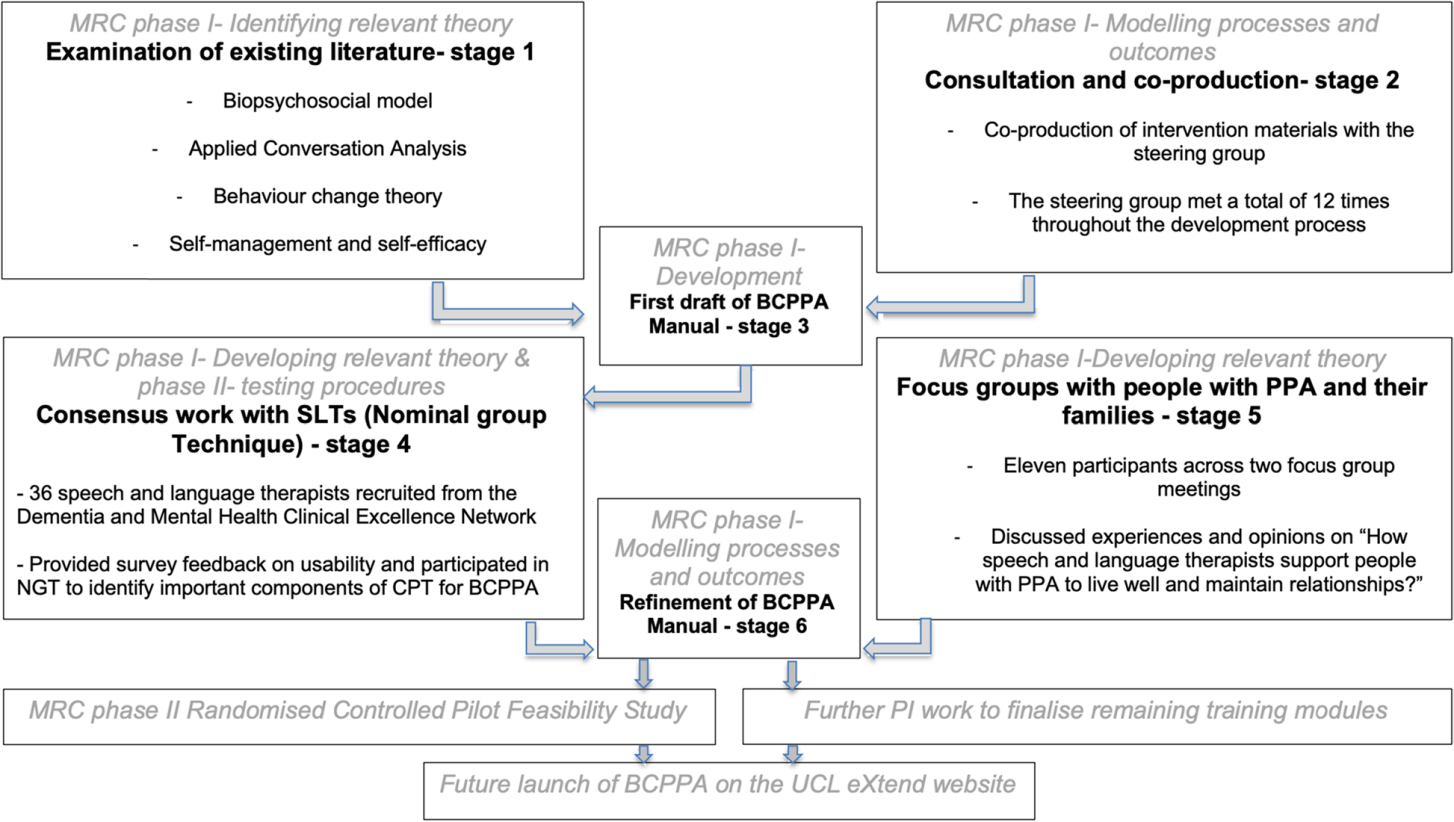
The six stages in the development BCPPA intervention and manual drafting, mapped on to the Medical Research Council framework for development of complex interventions

Intervention development activities were based on phases one and two in the Medical Research Council framework for development of complex interventions [29]. This comprised six stages including 1. examination of existing literature, 2. consultation and co-production work, 3. development of an initial draft, 4. consensus work with speech and language therapists, 5. focus groups with people with PPA and their families, 6. Refinement of the BCPPA intervention and manual in preparation for the randomised controlled pilot-feasibility study. Figure 1 demonstrates how these activities map onto the Medical Research Council guidance. Intervention development also followed the GUIDED guidelines for reporting for intervention development studies [30]. Further patient and public involvement work undertaken to finalise outstanding training modules identified as supplementary to the RCT will not be discussed here. The first author, A.V., an experienced speech and language therapist, led all stages. Work was undertaken over 2 years between 2016 and 2018.

### Recruitment

#### Consultation and co-production work (stage 2)

An opportunistic sample of people with PPA and their families, specialist speech and language therapists and neuropsychologists were invited to join the project steering group. A.V. emailed people who were known to her through clinical work and asked the facilitator of the PPA branch of the Rare Dementias Support Group based at UCL (https://www.raredementiasupport.org) to forward an invitation email to individuals in the support group, inviting them to participate.

#### Consensus work (Nominal Group Technique) with speech and language therapists (stage 4)

Speech and language therapists were recruited to participate in the Nominal Group Technique consensus study through the Royal College of Speech and Language Therapy Dementia and Mental Health Clinical Excellence Network, of which A.V. was a committee member. An advert was placed in the Royal College of Speech and Language Therapy clinical practice magazine (Bulletin) and via emails circulated to members inviting them to attend.

#### Focus groups with people with PPA and their families (stage 5)

People with PPA and their families who attend the PPA branch of the Rare Dementias Support Group at UCL were invited to participate in one of two focus group meetings held at an accessible venue on the university campus. The aim was to recruit eight people to each focus group, totaling 16 participants. To optimize opportunities for individuals with communication difficulties to contribute to discussion [31], group numbers were capped at eight participants. Potential participants who responded to the advert were contacted by A.V. on the telephone to judge if they met the inclusion criteria of a) a diagnosis or possible diagnosis of PPA/relative with such a diagnosis, b) the ability to communicate to participate in a focus group c) see and hear well enough to participate d) English as their language of daily use. Potential participants were excluded if they had a) a history of brain lesion or major head trauma, b) major physical illness or disability which could impact on participation. Criteria required.

### Examination of existing literature (stage 1)

Literature was selected following discussion with the research team to identify papers known to explore the theoretical underpinnings of interventions for dementia and CPT. The author then conducted searches of the reference lists of the articles to identify any other relevant articles. This included literature on existing models of dementia, principles of applied Conversation Analysis, behaviour change theory and frameworks for chronic disease self- management were explored. This informed the preliminary contents and focus of the intervention.

### Consultation and co-production work (stage 2)

There remains a lack of guidance on undertaking Public Involvement with people with communication difficulties [32] This work was therefore informed by information from the INVOLVE website [28] and bespoke advice from a co-author (K.S.) and expert on Public Involvement with people with stroke aphasia but modified to meet the needs of people in the group. Four people with PPA and their spouses, two expert speech and language therapists, a neuropsychologist and the group facilitator (A.V.) took part in 12 formal BCPPA Public Involvement steering group meetings. Public Involcement work to co-produce the BCPPA intervention materials and training modules was informed by feedback from people with PPA who had previously received CPT [32], research undertaken by A.V [10–12]. and research into the BCA program for people with stroke aphasia [33]. Discussion focused on identifying what distinct training modules would be required for the BCPPA training program and what the session plans and handouts would need to include for the manual. Once identified, a timeline for development was agreed and work undertaken to coproduce the content in steering group meetings. In order to support communication, steering group members were informed of the topic for discussion in advance of each meeting and invited to contribute in advance, during or after meetings using verbal, written or visual means, e.g. bringing photos, drawing pictures writing brainstorms or assembling and re-assembling draft materials.

### First draft of the manual (stage 3)

A draft of the BCPPA manual was developed using PowerPoint software. In order to upload these to the UCLeXtend website an online software package called Articulate was used to adapt the PowerPoint slides to an appropriate format. The work was undertaken with assistance from speech and language therapist researchers and four postgraduate researchers in speech and language sciences who were paid for their time.

### Consensus work (Nominal Group Technique) with speech and language therapists (stage 4)

The Nominal Group Technique was carried out at one of the Royal College of Speech and Language Therapy, Dementia and Mental health Clinical Excellence Network meetings. Draft one of the manual was made available to attendees (speech and language therapists). In order to gain an understanding of the clinical experiences and reality of speech and language therapists a qualitative research method was identified as appropriate. Speech and language therapists were encouraged to review the resource and pilot it with their clients. To ensure the BCPPA intervention reflected a consensus view of the most important components to include in a CPT intervention for people with PPA and their families a Nominal Group Technique method was chosen. Given that many of the speech and language therapists participating in the meeting had pre-existing professional relationships that could result in certain voices being represented over others in discussions, the Nominal Group Technique method was also chosen to provide opportunities to consider ideas and experiences equally yet allowing for clarification and discussion prior to rating [34].

Six weeks prior to attending the meeting speech and language therapists were sent an email inviting them to anonymously complete a 12-item feedback survey comprising all open questions (supplementary document 1), hosted online on the Google Forms platform. Survey questions were developed by A.V. in consultation with the steering group and included questions about speech and language therapists’ experiences and views on the content and format of the manual.

The Nominal Group Technique meeting itself comprised a two-stage ranking process commencing with a 90-min group session (stage one), followed by email consultation (stage two). Meeting facilitators (AV and SB) agreed the session plan and central question for discussion in advance (see supplementary document 2), in line with guidelines for conducting Nominal Group Technique meetings [14]. At stage two, results of the group session were circulated via email to all participants, providing information on scores and mean rankings for each item. As per guidelines for conducting Nominal Group Technique meetings [32], items describing the same ideas from the two groups were merged, following discussion and agreement between A.V. and S.B. Participants were asked to reply via email identifying and ranking their top eight items from this list (by placing a number from 1 to 8 to reflect which is most important - 8 and least important - 1). Following Nominal Group Technique guidelines [34], scores were tallied and mean rankings calculated to identify the top eight ranked items overall.

### Focus groups with people with PPA and their families (stage 5)

Two focus groups took place, to provide people with PPA and their families the choice of attending with or without partners. Discussion was guided by the question ‘How can speech and language therapists support people with PPA to live well and maintain relationships?’. The focus groups were jointly facilitated by A.V., alongside volunteer student speech and language therapists from UCL (one per focus group). A topic guide was co-produced with the BCPPA steering group and attendees of the PPA branch of the Rare Dementia Support Group at UCL (see supplementary document 3).

Focus group discussions were video recorded and transcribed by UCL student speech and language therapists (using transcription guidance [35]). Given the researchers objectives to understand the lived experiences of people with PPA and their families, and gather opinions from them, qualitative methods employing a realist approach to reflexive thematic analysis was undertaken [36, 37]. Initial codes were generated by systematically coding interesting features (phase 2), collating these into potential themes (phase 3) and reviewing them in relation to the coded extracts (phase 4). Potential themes were refined to generate definitions and names (phase 5), further inspected to identify and report any additional key elements (phase 6). In addition, to improve reliability of analysis, four speech and language therapist researchers with experience of thematic analysis independently extracted data from a randomly selected section of transcript, discussed and reached agreement on the coding of themes arising from the data.

### Refinement of the BCPPA manual (stage 6)

Results of work in stages 4 and 5 of intervention development were presented to the project steering group. Refinements were jointly identified and agreed by the group members.

## Results

### Examination of existing literature (stage 1)

Existing literature comprising the bio-psychosocial model of dementia, applied Conversation Analysis, behaviour change theory and self-management and self-efficacy theory was examined.

### Bio-psychosocial model of dementia

The bio-psychosocial model [38] proposes that there are factors other than the organic causes of dementia that influence the nature and speed of deterioration in daily functioning. These include some factors that are fixed, such as PPA variant, that cannot be changed. The BCPPA manual therefore provides practice tasks, to maximise generalisation for people with semantic PPA, for whom this is more difficult than those with non-fluent PPA. Tractable factors, such as the way a CP interacts with a person with PPA, may be amenable to change and are directly targeted in the BCPPA intervention. Adaptive mechanisms used by the CP, such as multiple questions or test questions, may result in the person with PPA feeling incompetent [13]. On the other hand, the use of gesture and enactment (whole body gesture and pantomime) by a person with PPA when they are having difficulty retrieving a spoken word [39] could be described as an effective coping strategy. The BCPPA intervention seeks to take account of fixed factors whilst targeting tractable factors to support the dyad (person with PPA and their CP) achieve their potential function.

### Applied conversation analysis

Conversation Analysis is an approach to the study of human social interaction through the analysis of spontaneous, naturally occurring talk [40]. A number of Conversation Analysis informed stroke aphasia intervention studies and clinical resources have been developed [41] such as Supported Conversation for adults with Aphasia [18] Supporting Partners of People with Aphasia in Relationships and Conversation [41] and BCA [42]. These have in common the analysis of video recordings of natural conversations between the person with aphasia and their CP, and providing these as video feedback, as a foundation for targeting therapy [41]. The speech and language therapist (who typically delivers such an intervention) analyses 10-15 min video-recorded interaction to identify behaviours resulting in conversational breakdown, known as barriers, and ways in which members of a dyad successfully resolve or circumvent troubles to maintain interaction, known as facilitators. The aim of video feedback is to increase awareness in one or both members of the dyad of the impact of their behaviours, and jointly agree on goals for therapy. Once the goals of therapy are agreed upon, a process of practice, through supported conversations, role play and reflection, is commonly employed [41]. The BCPPA intervention is informed by this well-described [43], CA-underpinned approach to CPT.

### Behaviour change theory

Recognising conversational barrier behaviours in video recordings of oneself and setting a goal to cease these, or adopt facilitative strategies instead, does not guarantee that a change in behaviour will occur [44]. Behaviour change theory, specifically the COM-B model [33] accounts for an individual’s behaviour change as the product of three equally weighted components namely Capability, Opportunity and Motivation. Researchers examined video recordings of Conversation Analysis-underpinned CPT being delivered to people with stroke aphasia and their CPs [45] and used the COM-B model [33] to identify the essential change processes and the core procedures that serve them [46]. The BCPPA intervention incorporates the seven core mechanisms that have been identified as essential to behaviour change in a CPT [45], specifically the processes to motivate change and those that embed changes (See supplementary material 4).

### Self-management and self-efficacy

Central to self-management is the concept of the client as an active participant whose current status is influenced not only by diagnosis but by psychological responses and experiences. This implies interventions should address the ability to self-manage daily activities and the emotional journey, not just medical symptoms [46, 47]. Taking action to accomplish a plan to self-manage their condition is more likely to succeed if a person has the confidence or self-efficacy to achieve it [48]. Self-efficacy is a mechanism that directs behaviour change, for if one feels in control of a behaviour it becomes easier to make a change to it [49]. Five core self-management skills and four key self-efficacy mechanisms have been highlighted for inclusion in speech and language therapist interventions with people with progressive communication difficulties [48] and these have been considered in the development of the BCPPA intervention (see supplementary file 4).

### Consultation and co-production work with the steering group (stage 2)

Decisions made included:

1. Identification of seven subjects to form distinct training modules within the BCPPA program. Table 1 provides an overview of the learning objectives and how these were co-produced. The three modules required for the phase II NHS based randomised controlled pilot-feasibility study (Module 3: How to make a video, Module 4: What to target in therapy and Module 5: the BCPPA therapy) were prioritised for development over the four only needed for the future general release of the online BCPPA program. Table 2 provides an overview of the content of these three modules.
2. Development of a topic list, for Module 3: How to make a video, to support participants when making video recordings of their own conversations.
3. Distillation of the components of the eight BCA sessions into four 1- h BCPPA sessions (the duration agreed-upon by speech and language therapists as feasible [11, 12])

**Table 1:** Learning objectives and timeline for development of the BCPPA training modules including the therapy program

BCPPA training modules	Learning objectives for speech and language therapists accessing the module	Module components	Development timeline
Module 1: What is PPA?	To explain what PPA is according to: - People with PPA and their relatives who have worked on this module, - Speech and language therapists working in the area - The research literature in this area	Co- produced with steering group. References selected by steering group.	Prior to launch of online BCPPA program
Module 2: What is conversation training?	To explain what conversation training is to clients, based on video recorded interviews with: - Speech and language therapists working in the area - People with PPA and their relatives who have worked on this module.	Co- produced with steering group Video clips planned, filmed and selected by steering group	Prior to launch of online BCPPA program
Module 3: How to make a video	• To have an appropriate tool available to gain consent for the purpose of videoing of a couple in conversation with one another to be used in the conversation training intervention, BCPPA. • To be aware of the Mental Capacity Act (2005) and how this will impact on consent. • For speech and language therapists to be supported to make and store videos, in line with the data management guidance and policies of their local organisation, of conversation between a client and their conversation partner for the purpose of the BCPPA intervention. • To be able to set up an optimal environment for the purposes of making a video for the BCPPA intervention	Co-produced work with the steering group included: a topic sheet to support participants in identifying what to discuss during video recording, example consent forms, video samples and formatting of module.	Prior to Phase II RCT Feasibility Pilot Study
Module 4: What to target in therapy	To understand the three stages of the goal setting process: 1) Identification of facilitators and barriers from pre-therapy videos 2) Selection of suitable video clips of appropriate length and focus to show clients, and 3) Negotiation of goals with a person with PPA and their conversation partner	Co- produced work with people with PPA included: video samples and formatting of module.	Prior to Phase II RCT Feasibility Pilot Study
Module 5: BCPPA therapy	To deliver the four **synchronous** BCPPA therapy sessions, supporting people with PPA and their communication partners (as a dyad) to: - Understand concept of barriers and facilitators in conversation and consider thesis briefly in relation to their own conversation - Identify barriers and facilitators in their own conversation - Set goals for therapy based on this discussion - Practice conversation using the strategies identified during goal setting - Problem solve any issues that have arisen in using identified strategies in conversations outside of therapy sessions - Consider planning for future changes in communication	Co- produced work with steering group included: Therapy handouts for sessions 1 and 4, therapy activities for session 3, video samples and formatting of module.	Prior to Phase II RCT Feasibility Pilot Study
Module 6: Measuring it	• To consider what options are available for measuring outcomes for BCPPA; • To think about the pros and cons of different outcome measures; • To consider how to use outcome measures in clinical practice.	Co-produced with speech and language therapists working with people with PPA (local collaborators who participated in the Phase II RCT Feasibility Study)	Prior to launch of online BCPPA program
Module 7: Useful Resources	• To learn about some activities people with PPA enjoy; • To find out about some useful websites and resources; • To have thought about what has been useful in your therapy.	Co-produced with steering group Online resources selected by steering group	Prior to launch of online BCPPA program

The language used for module titles and learning objectives reflects vocabulary selected by the steering group during co-production and was felt appropriate and accessible for the target audience (clinical speech and language therapists)

*PPA* Primary Progressive Aphasia, *BCPPA* Better Conversations with Primary Progressive Aphasia, *RCT* Randomised Controlled Trial

**Table 2:**
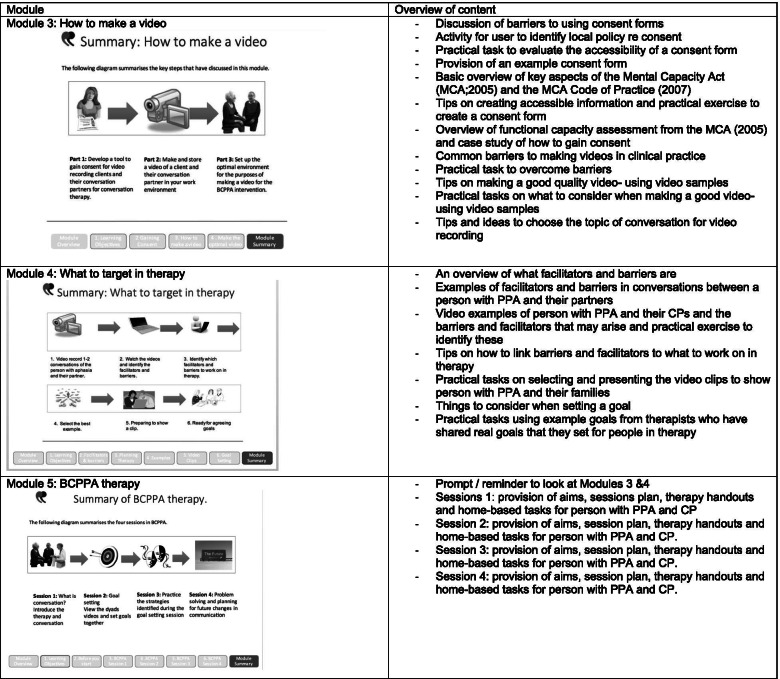
Overview of content for the first draft of the BCPPA manual (Modules 3, 4 & 5)

*PPA* Primary Progressive Aphasia, *MCA* Mental Capacity Act, *CP* Communication Partner, *BCPPA* Better Conversations with PPA

### First draft of BCPPA manual (stage 3)

Module 5: the BCPPA therapy, hosted the BCPPA manual comprising session plans, session handouts and home-based tasks for each of the four BCPPA intervention sessions. The session plans identified intervention components as either core or non-essential components that can be tailored to an individual’s needs.

The draft manual was evaluated by the steering group to ensure information was presented in an accessible way. This included decisions on images and formatting.

The first draft of the manual was uploaded to a secure area on the UCLeXtend website and made available to speech and language therapists participating in the stage 4 consensus work via a bespoke URL. It was not publicly accessible.

### Consensus work (Nominal Group Technique) with speech and language therapists (stage 4)

#### Demographics and characteristics of speech and language therapist participants

Thirty-six speech and language therapists took part. Of these, 17 had completed the pre- Nominal Group Technique meeting survey, 22 had viewed the first draft of the BCPPA manual and training program prior to attending, and two had been able to use the BCPPA manual with a client with PPA. Table 3 presents speech and language therapist participant demographics and their familiarity with the BCPPA manual and training program. Following the meeting, 20 of the 36 participants completed the final Nominal Group Technique ranking task by email.

**Table 3:** Demographics of speech and language therapists who participated in the Nominal Group Technique meeting and their familiarity with the BCPPA program

	Speech and language therapist participants (*n* = 36)
Gender (m:f)	2:34
Years practicing as a speech and language therapist (mean and range)	12.5 (0–21)
Number of clients with PPA seen in clinical career (mean and range)	9 (0–20)
BCPPA modules viewed online prior to meeting:	
None but knows of BCA	1
None	11
Module 3 How to make a video Module	22
4 What to target in therapy	21
Module 5 BCPPA therapy	22

*m* Male, *f* Female, *PPA* Primary progressive aphasia, *BCA* Better Conversations with Aphasia program, *BCPPA* Better Conversations with PPA program

#### Pre- Nominal Group Technique meeting survey

**Fig. 2:**
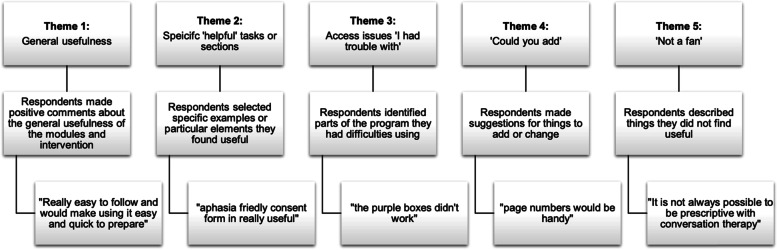
Themes identified from survey responses in Stage 4 consensus work

When asked what surprised them when they first accessed the online BCPPA program five of 17 respondents (29%) commented on there being a lot of detail. Five respondents (29%) described the program as clear, easy to use and accessible; one person highlighted the comprehensive and detailed step by step guidance. A further four respondents (24%) stated that they were unsurprised by the BCPPA program, given their familiarity with the BCA program on which BCPPA is based. Respondents provided feedback on the BCPPA program including the most useful aspects (17, 100%, respondents), formatting (16, 94%, respondents), additions or changes (14, 82%, respondents) and the least useful aspects of the program (10, 60%, of respondents). Five themes arose from these data: 1. General usefulness; 2. Specific ‘helpful’ tasks or sections; 3. Access issues, ‘I had trouble with’; 4. ‘Could you add’; 5. ‘Not a fan’. These themes are illustrated with quotes in Fig. 2. Notably, access issues were generally related to glitches in the program, though some local NHS browser systems posed restrictions.

#### Nominal Group Technique

After two iterations of consensus work with speech and language therapists, focused on the question “*What components of the BCPPA therapy sessions are important for people with PPA and their conversation partners*?”, eight components were identified, and ranked in order of importance, see Table 4.

**Table 4:** Final eight ranked components identified as important for the BCPPA program, from two stage Nominal Group Technique consensus work

1	Use of video feedback to identify facilitators versus barriers in conversation when focusing on people’s strengths as well as areas of potential breakdown
2	Tailored and person centred: - goals, - conversational topics, - strategies - practice opportunities
3	Emphasising a focus on getting message across rather than a perfect interaction
4	Focusing individual attention on non-verbal communication strategies such as body language, gesture, facial expression and other methods of total communication.
5	Recognising and building on current communication strengths.
6	Working with both the person with PPA and the CP together.
7	Providing opportunities to practice strategies and get feedback from the speech and language therapist.
8	Providing an opportunity to discuss their communication difficulties

*PPA* Primary Progressive Aphasia, *CP* Communication Partner

### Focus groups with people with PPA and their families (stage 5)

#### Demographics of participants

Thirteen participants, six people with PPA and seven family members, responded to the advertisement. All were eligible and agreed to participate but one couple withdrew the day before the focus group due to a conflicting commitment. The remaining 11 participants attended two focus groups (NB: these were mixed groups, whereby people with PPA and their CPs attended together, alongside some CPs and people with PPA who attended independently, group 1: seven participants; group 2: four participants). Participants with PPA represented all three variants, and atypical mixed variants. Demographic information is outlined in Table 5.

**Table 5:** Demographic information for focus group participants

	Person with PPA (PwPPA) and communication partner (CP)	PPA variant	Time since symptom onset	Time since diagnosis
Focus Group 1:	PwPPA (m) + CP (f)	lvPPA	4 years,	2 years
	PwPPA (f) + CP (m)	Mixed	3 years	2 years
	CP (f)	(Mixed)	(9 years)	(4 years)
	PwPPA (f) + CP (m)	nfvPPA	5 years	4 years
Focus Group 2:	PwPPA (m)	lvPPA	4 years,	1 year
	PwPPA (f) + CP (m)	svPPA	5 years	4 years
	CP (m)	(Mixed)	(8 years)	(5 years)

*PwPPA* Person with primary progressive aphasia, *CP* Communication partner, *lvPPA* Logopenic variant primary progressive aphasia, *svPPA* Semantic variant primary progressive aphasia, *nfvPPA* Non-fluent agrammatic variant primary progressive aphasia

#### Themes arising from the focus groups

**Fig. 3:**
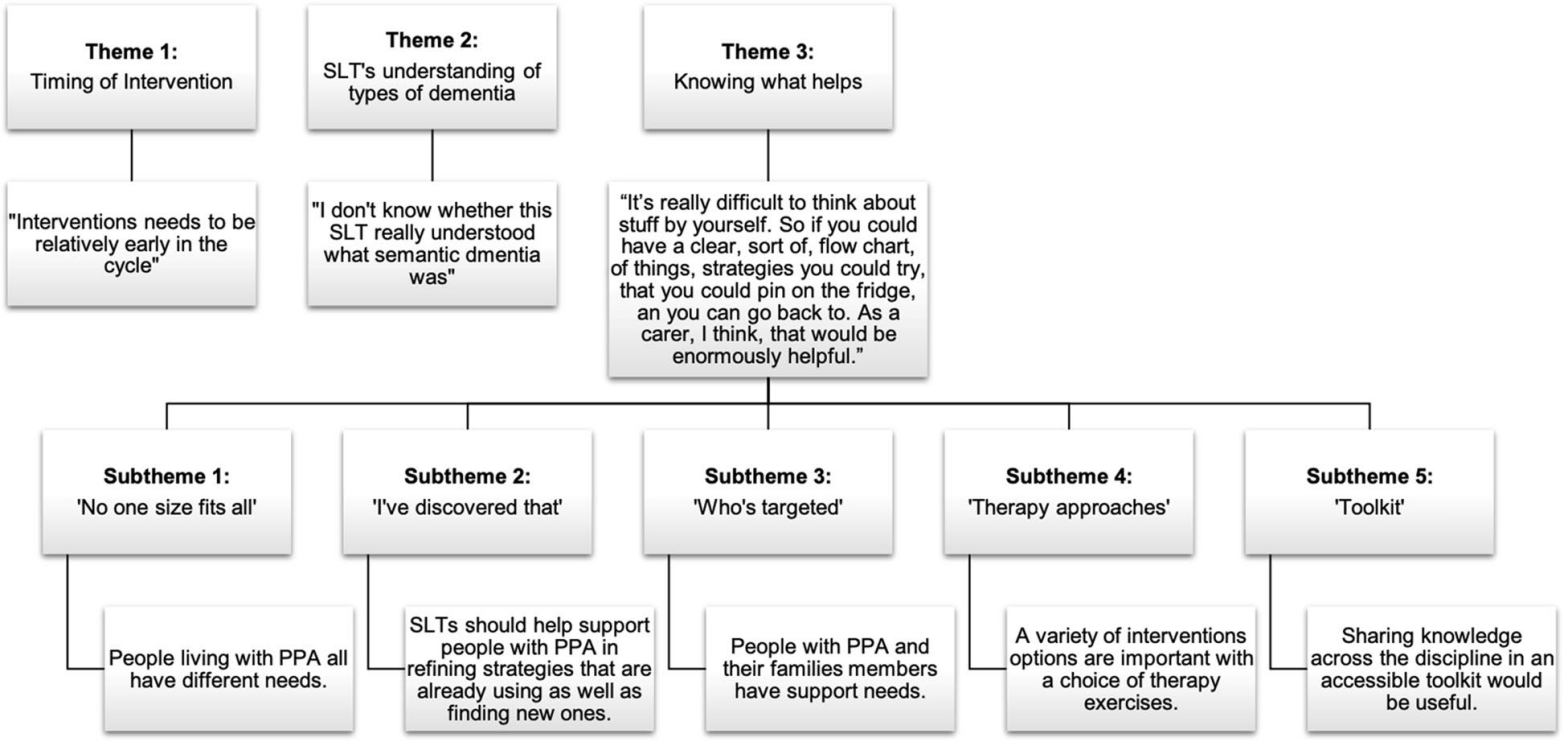
Themes and subthemes arising from focus groups with people with PPA and their CPs

Three overarching themes emerged: 1) Timing of intervention, 2) speech and language therapists’ understanding of types of dementia, and 3) Knowing what helps. Theme 3 encompassed five further subthemes: ‘No one size fits all’, ‘I’ve discovered that’, ‘who’s targeted’, ‘therapy approaches’ and ‘toolkit’. All themes and subthemes are presented in relation to illustrative units of data in Fig. 3.

### Refinement of BCPPA manual (stage 6)

Refinements for the BCPPA manual are presented in Table 6. The refined BCPPA program was consequently made available to participating local speech and language therapist collaborators on UCLeXtend as part of their training in preparation for delivering the intervention during the randomised controlled pilot-feasibility study. The final intervention is described in detail, using the Template for Intervention Description and Replication (TiDIER), in the authors PhD thesis which this paper is based on [50], and a published protocol for study which remains currently underway [51]. Further to this, the project steering group made plans to continue working to co-produce the remaining four modules, in anticipation of a future launch of the BCPPA program. This paper is based on work from the authors PhD thesis.

**Table 6:** Refinements for BCPPA manual and intervention

Decisions made	Examples of refinements made
Provide more options on strategies and practice activities in the intervention materials.	Addition of Home based task 2: Strategies to help turntaking and expansion of session plan 3 to include a list of 11 optional additional strategy practice ideas based on ideas collated from speech and language therapists, people with PPA and their families and a review of manuals for stroke aphasia CPT manuals.
Provide more information on resources and other services.	Expansion of session plan 4 to include a list of resources and other services for speech and language therapists making recommendations for the future.
Develop video examples of the intervention being delivered.	Addition of video recordings of conversation breakdown and intervention being delivered inserted to Module 5: The BCPPA therapy. These included: Session 1: Video examples of Keith and Rose watching videos of themselves and the speech and language therapist facilitating them to identify barriers and facilitators. Session 2: Video examples of Keith and Rose goal setting with the speech and language therapist. Session 4: Video example of Keith and the speech and language therapist discussing a difficult subject around future planning.
Include more testimonies from people with PPA in Module 1: What is PPA and Module 2: What is communication partner training?	Use of quotes to illustrate experience of communication facilitators and barriers in Module 4: What to target in therapy.
Provide more information on how PPA impacts on daily communication.	Refinement of Session 1. Handout 1. How does conversation work? And addition of Session 1. Handout 2. What can go wrong in conversations? in co- production with project steering group.
Provide a summary sheet including suggestions for future changes on one handout at the end of the intervention.	Addition of summary handout for session 4: Handout 6: Your strategies

*BCPPA* Better Conversations with Primary Progressive Aphasia, *PPA* Primary progressive aphasia

## Discussion

The BCPPA manual and training program were developed using the framework described in the Medical Research Council guidelines for development of complex interventions [29]. The intervention content is underpinned by the bio-psychosocial model of dementia, applied CA, behaviour change theory, and self-management and self-efficacy literature. Consultation and co-production work with a project steering group made up of people with PPA and their family members provided the first draft of the BCPPA manual and training program. Consensus work using a Nominal Group Technique with practicing speech and language therapists and focus groups with people with PPA and their families, identified further refinements. These included additions to the manual, and modifications to improve access to and use of the materials within the modules.

Speech and language therapists report seeing people with PPA in their clinics who feel incompetent in conversations, whilst their CPs feel helpless to support them in these situations [52]. Addressing this by exploring meaningful strategies to maintain conversation via CPT that involves both a person with PPA and their CP has been recommended by expert speech and language therapists [26]. Currently, speech and language therapists delivering CPT to people with PPA and their CPs report using tools designed for people with stroke aphasia because there are no PPA-specific materials [11, 12]. The BCPPA manual and training program address this gap in the speech and language therapists’ “toolkit” (described as such by participants in the focus groups) of interventions for PPA, and provides an evidence based, manualised training resource designed by and for people with PPA and their CPs.

### Strengths and limitations

Drawing on the best available evidence and appropriate theory to develop the BCPPA manual, in accordance with Medical Research Council guidance [29], should increase the likelihood that components of the intervention result in behaviour change. Extensive use of theory has been associated with larger effect sizes in a review of online behaviour change interventions [53]. This work has involved new research with those targeted by the intervention as well as those delivering it.

There are, however, some methodological limitations. Nominal Group Technique does not allow for anonymisation in the way that other consensus methods such as Delphi do, and can thus bias the responses of participants. Unfortunately, only 20 of the 36 participants who attended the original meeting completed the final Nominal Group Technique ranking task by email. These numbers may be associated with the fact that some participants did not have experience working with people with PPA. The Nominal Group Technique did nevertheless, provide a method of involving large participant numbers and incorporating mathematical voting techniques to aggregate group judgements equally [34]. Despite only 12 of the participants who attended the Nominal Group Technique meeting having viewed the modules beforehand, making the intervention manual available enabled scrutiny of its practicality for clinical practice in anticipation of the phase II NHS based randomised controlled pilot-feasibility study. Notably, only two males were recruited to the Nominal Group Technique, though this is generally representative of the current speech and language therapy community [54]. Despite being a useful method for eliciting participant’s genuine and honest opinions, a focus group can be a challenging communication environment [55]. The role of the speech and language therapist facilitator and the student speech and language therapist co-facilitators was to mitigate this by enabling participants to contribute to discussion. The option to attend with CPs to support communication was also provided, but instead participants prioritised the convenience of meeting dates and times. Given the steering group was established a number of years prior to the recently published practice standards for Public Involvement [56] it is likely that the methods employed may have limited the effectiveness of the co-produced work. Some have criticised the steering group model for consulting with only a small number of individuals. There were only three couples with PPA in this group and that may have limited its value. PPA is, however, a relatively rare condition and people were approached to reflect the known diversity within the condition. Additionally, new members were sought when others withdrew due to disease progression, and the author sought to gather perspectives of other people and their families through individual telephone contact. Despite approaching professionals from other disciplines, including medicine and social work, interested individuals were not able to attend steering group meetings. The author was able to consult with the research team, including neurology colleagues, to gather feedback and ideas.

A manualised approach enables standardised delivery of the intervention for a future trial. Given that speech and language therapists in clinical practice may have limited experience of working with people with PPA [10, 11], this helps to maximise ease and fidelity of delivery for future implementation. However, a manualised intervention may limit the potential to tailor an intervention to individual clients, for example by deciding not to use video recording or by delivering the intervention to a person accompanied by two CPs. Person-centred components have been identified as important for functional communication interventions for people with PPA, and have been highlighted as important for behaviour change [45, 46]. The development of this intervention took behaviour change theory into account and embedded the core processes and mechanisms that had been identified in previous CPT research as essential components. These were clearly signposted in the manual and distinguished from non-essential components that were amendable to tailoring. Furthermore, expecting four 1-h therapy sessions to result in a change may seem ambitious. However, the decision on dosage was made based on the average number of sessions that speech and language therapists reported having available to deliver functional communication interventions for PPA [11]. Developing an intervention that meets this requirement increases the chance of implementation.

## Conclusions

The six-stage process of development included a review of existing literature, and consultation and co-production with the project steering group to develop an initial draft. Consensus work undertaken with speech and language therapists and focus groups with people with PPA and their families identified further refinements. The BCPPA manual was refined in preparation for a phase II NHS based randomised controlled pilot-feasibility study which is currently underway [51].

## Supplementary Information

**Additional file 1: 1.** Survey instructions and questions disseminated to SLTs prior to CEN study day. **2.** Session plan for Nominal Group Technique with SLTs. **3.** Topic guide for focus groups with people with PPA and their families. **4.** Seven core mechanisms underpinning conversational behaviour change. **5.** Self-management skills and self-efficacy mechanisms.

## Data Availability

Not applicable.

## Abbreviations

PPA: Primary Progressive Aphasia
CP: Communication Partner
CPT: Communication Partner Training
BCPPA: Better Conversations for Primary Progressive Aphasia
NHS: National Health Service
