# Supplementary material for: Using the Medical Research Council framework and public involvement in the development of a communication partner training intervention for people with primary progressive aphasia (PPA): Better Conversations with PPA
Source: BMC Geriatr. 2021 Nov 15;21:642. doi: 10.1186/s12877-021-02561-8 (PMC8591912; doi:10.1186/s12877-021-02561-8)
Supplement: Supplementary file 1 — Additional file 1: 1. Survey instructions and questions disseminated to SLTs prior to CEN study day. 2. Session plan for Nominal Group Technique with SLTs. 3. Topic guide for focus groups with people with PPA and their families. 4. Seven core mechanisms underpinning conversational behaviour change. 5. Self-management skills and self-efficacy mechanisms. [file 12877_2021_2561_MOESM1_ESM.docx]

Supplementary Materials:

1. Survey instructions and questions disseminated to SLTs prior to CEN study day
2. Session plan for Nominal Group Technique with SLTs
3. Topic guide for focus groups with people with PPA and their families
4. Seven core mechanisms underpinning conversational behaviour change.
5. Self-management skills and self-efficacy mechanisms.

1.Survey instructions and questions disseminated to SLTs prior to CEN study day

The BCPPA program is in the process of being developed and tested. In order to keep refining we need your expert critique. We want this to be a useful program with things that will help in clinical practice. Please do be as honest as possible – this is what is most useful.

Your answers WILL be anonymous!!!

1. What was surprising to you when you first accessed BCPPA?
2. List one positive and one negative aspect of Module 3: How to make a video?
3. List one positive and one negative aspect of Module 4: What to target in therapy?
4. List one positive and one negative aspect of Module 5: BCPPA therapy
5. Is there anything you would delete, add or change?
6. Have we missed anything important?
7. Are any materials less useful?
8. What do you feel is most useful in the entire BCPPA program so far?
9. Is the format usable (i.e. the separate modules, with session plans, sessions handouts and home based tasks as downloadable documents) - what would you change?
10. Is the program deliverable for people with PPA- if not why not?
11. We will be developing some other modules to support these- is there anything we should include?
12. Any other comments:

2. Session plan for Nominal Group Technique with SLTs

1. Introduction:

Facilitators introduce themselves to the group.

AV provides a brief summary of the BCPPA program content and processes for the meeting.

1. Attendees separate into two groups, each seated in a semi-circle
2. Research question to be presented:

What components of the draft BCPPA therapy sessions are important for people with PPA and their conversation partners?

1. Facilitators hand out sheets for 10 minutes of silent work. Participants asked to write down:

a) demographic information (gender, years of experience, number of people with PPA seen in their career),

b) Ideas to answer research question.

1. Participants to be asked to share ideas in turn within their group. Facilitator records each idea on a flip chart until all ideas are listed. Ideas are recorded verbatim. Each idea will be given a number.
2. Once recorded each idea will be revisited in turn and the following question asked “Does anyone have any questions about this point and what it means?” A maximum of 3 minutes will be spent clarifying each point. Participants to be prompted that this time is for clarification only, not to provide opinions.
3. Facilitators to hand out eight cards to each participant and ask them to spend 10 minutes in silence choosing the 8 most important items from the flip chart and writing one on each card. Once finished participants o be asked to spend another 10 minutes in silence ranking their choice of eight items from 1-8 (8 being the most important and 1 being the least important).
4. Once completed participants place their anonymous rankings in envelopes and return them to the facilitator.
5. Finish:

All attendees to be thanked for their participation. All attendees to be informed that the results from the two groups would be collated and circulated for final ranking via email.

3: Topic guide for focus groups with people with PPA and their families

This topic guide has been designed, reviewed and reformulated with advice from the project steering group and service users through the PPA branch of the Rare Dementia Support Group at UCL.

Duration of focus groups including refreshments: 2 hours

1. Introduction of group facilitators, final check that consent paperwork has been completed.

2. Introduction to research topic (use title), reminder re confidentiality and respecting others opinions and plan for today

3. (start video) Invite group members to introduce themselves:

“First of all I would like to invite you all to introduce yourselves to the group by telling us your name and how PPA affects your life, perhaps X could start us off?”.

(30 minutes)

1. Question to guide discussion:

“How can speech and language therapists support people with PPA to live well and maintain relationships?”

Prompts:

What do you no longer talk about? and What would you like to talk about?

What interventions/resources have you had that have been useful / successful?

(45 minutes)

6. Close and thank you, advise group on how data will be used and when they will receive a summary of the focus group findings.

(10 minutes)

4. Seven core mechanisms underpinning conversational behaviour change.

| Opportunity | 1. | Change in conversation support for people with aphasia’s strategies, whereby the CP prompts their partner to use gesture for example. |
| --- | --- | --- |
| Capability | 2. | Increased awareness of own behaviour, such as watching video recordings to facilitate reflection of the impact of behaviours on others. |
|  | 3. | Replacing barriers with facilitators, such as replacing test questions with clarification questions. |
|  | 4. | Increased ease at implementing strategies through home based practice tasks between intervention sessions for example. |
| Motivation | 5. | Changed expectation of behaviour’s impact, such as trialling strategies to demonstrate success. |
|  | 6. | Changed priorities for conversation, by, for example, discussion on the purpose of conversation i.e. eliciting accurate verbal communication versus interaction through any mode of communication. |
|  | 7. | Changed perception of success in conversation, for example by recognising success, which in turn boosts confidence. |

5. Self-management skills and self-efficacy mechanisms.

| 1.Owning problem-solving skills: In BCPPA the SLT works as a facilitator, guiding the person with PPA and their CP to identify problems in their conversations, analysing video clips of their own conversations, and how they will address these, by setting their own meaningful goals for therapy.  2. Decision-Making: The person must have a good understanding of their condition and what to expect as it progresses. The BCPPA intervention provides opportunities to discuss how the progression of PPA will lead to continued changes in conversations.  3. Resources: Arguably a CP is one of the most useful resources for a person with PPA in conversation, thus including them in the BCPPA intervention should maximise the utility of this resource.  4. Relationships with healthcare providers: The BCPPA intervention includes the provision of information on future contact with SLT and support services.  5.Taking action: A large component of BCPPA includes practicing strategies, both within the therapy sessions and outside of therapy as part of home based tasks.  Yorkston et al's (2017) self-efficacy mechanisms are:  1. Performance mastery: BCPPA provides the opportunity for people with PPA and their CPs to practice strategies throughout the intervention period, strengthening their belief in their own ability to use these strategies.  2.Finding models: Including examples of experiences of other people with PPA and their CPs in the intervention materials provides models for people participating in the intervention.  3.Reinterpreting symptoms: By focusing on conversation as a dynamic flow of communication between two people, a person with PPA and their CP are re-orientated to understand that there may be multiple contributing factors impacting on their conversations.  4. Social persuasion: Involving both a person with PPA and their CP throughout the intervention enables mutual persuasion. |
| --- |
